# Do Antioxidant Phytochemicals Play a Role in Neurodegenerative Disorders? The Case of Polyphenols

**DOI:** 10.3390/nu14224826

**Published:** 2022-11-15

**Authors:** Justyna Godos

**Affiliations:** Department of Biomedical and Biotechnological Sciences, University of Catania, 95123 Catania, Italy; justyna.godos@gmail.com

In recent decades, numerous studies provided consistent and convincing evidence that the adoption of healthy plant-based dietary patterns is a valuable strategy to reduce the risk of most non-communicable diseases [1,2]. Several factors, including the scarce intake of plant-derived foods, accompanied by the overconsumption of animal products, have been shown to be responsible for mortality and years of life lost due to cardio-metabolic diseases and certain cancers [3,4,5,6,7,8,9]. Among the most important beneficial factors, the intake of essential nutrients and fiber is certainly the most studied and, quite convincingly, a responsible factor for the benefits of adopting primarily plant-based dietary patterns [10]. However, emerging evidence suggests that other factors, such as the dietary content in polyphenols, might be as important as the aforementioned dietary features in the prevention of cardio-metabolic disorders and prolonging lifespan [11,12]. A key characteristic behind the benefits of these heterogeneous groups of molecules is their chemical diversity, which provides a variety of mechanisms of action resulting in a multitude of potential health targets [13].

Among the most recent areas of research, the potential action of polyphenols in the central nervous system and related diseases, including neurodegenerative disorders, is of major interest [14]. Diet has been related to important changes in brain health, with polyphenols and gut microbiota being potentially involved in the process [15]. The suggested mechanisms of action of polyphenols in counteracting neurodegenerative disorders lay the ground for the physiopathological mechanisms promoting such conditions. In fact, it has been hypothesized that most neurodegenerative diseases may be promoted or aggravated by neuroinflammation, resulting in the alteration of certain brain areas, structures, and functionality [16]. Inflammation leads to the production of inflammatory mediators (i.e., cytokines) with neurotoxic effects [17]. At the cellular level, the oxidative stress generated by the activation of inflammatory pathways, leading to mitochondrial damage and dysfunction, seems to potentially have an important role in neuronal degeneration and the promotion of neurodegenerative diseases [18]. In this context, environmental factors may influence the establishment of the neuroinflammatory mechanisms that underlie one of the pathogenetic processes of neurodegenerative diseases [19]. Among others, researchers have provided growing mechanistic evidence that potentially explains how diet may affect the human brain [20]. Concerning polyphenols, several mechanisms have been reported that describe the potential effects of such molecules on the human brain.

In our Special Issue entitled “Polyphenols and Polyphenol-Rich Foods in Neurodegenerative Disorders”, several articles are published that provide further evidence on the potential role of polyphenols in brain health. The study of Al-Musharaf et al. [21] investigated the association between dietary polyphenols and sleep quality in 92 normal-weight and obese young women, reporting that only higher polyphenol intake and homeostasis model assessment of insulin resistance (HOMA-IR) were inversely associated with poor sleep quality (with no significant findings for other nutritional-related biomarkers). Another two studies provided insights from animal models on Parkinson’s and Alzheimer’s diseases. The study of Witucki et al. [22] showed the neuroprotective effects of cranberry juice treatment in a rat model of Parkinson’s disease by affecting various mechanisms, including reducing α-synuclein accumulation, Bax and cleaved/active caspase-9 expression, and normalizing cytochrome c level. The other study on animal models from Igarashi et al. [23] involved senescence-accelerated mice in which matcha and decaffeinated matcha were administered, resulting in the regulation of the expression of a several of proteins potentially involved in Parkinson’s and Alzheimer’s diseases. The other studies published in the Special Issue provided two important overviews of tea polyphenols, including phenolic acids, in brain aging [24] and cognitive disorders [25], highlighting the description of the mechanisms underlying the inhibition of neuroinflammatory processes and the prevention of degenerative effects in the aging brain.

Overall, the Special Issue provides interesting insights on the role of certain polyphenols, especially underrated polyphenols such as phenolic acids, and brain health for the prevention of neurodegenerative diseases. Additional studies are encouraged for submission to further explore this research topic and broaden the field of exploration of polyphenols on the benefits for the human brain.
